# Implementation and acceptance of pharmacists’ prescribing of human immunodeficiency virus (HIV) pre-exposure prophylaxis (PrEP)

**DOI:** 10.1177/17151635251355277

**Published:** 2025-08-22

**Authors:** Mackenzie d’Entremont-Harris, Tasha Diana Ramsey, Kathleen MacNabb, Andrea Murphy, Andrea Bishop, Jennifer E. Isenor, Deborah V. Kelly, Yazid N. Al Hamarneh, Matthew Lee, Abbey Ferguson, Kirk Furlotte, Lisa Woodill, Todd Hatchette, Kyle John Wilby

**Affiliations:** Pharmacy Department, Nova Scotia Health, Halifax, NS; Pharmacy Department, Nova Scotia Health, Halifax, NS; Faculty of Health, College of Pharmacy, Dalhousie University, Halifax, NS; Pharmacy Department, Nova Scotia Health, Halifax, NS; College of Pharmacy, Psychiatry, Nursing, Dalhousie University, Halifax, NS; Nova Scotia College of Pharmacists, Halifax, NS; College of Pharmacy, Faculty of Health, Dalhousie University, Halifax, NS; School of Pharmacy, Memorial University, St. John’s, NL; Faculty of Medicine and Dentistry, University of Alberta, Edmonton, AB; Halifax Sexual Health Centre, Halifax, NS; Halifax Sexual Health Centre, Halifax, NS; Community-Based Research Centre, Vancouver, BC; The Pharmacy Association of Nova Scotia, Dartmouth, NS; Department of Pathology, Dalhousie University, Halifax, NS; Department of Pathology and Laboratory Medicine, Nova Scotia Health, Yarmouth, NS; College of Pharmacy, Faculty of Health, Dalhousie University, Halifax, NS

## Abstract

**Background:**

Pre-exposure prophylaxis (PrEP) for human immunodeficiency virus (HIV) is highly effective at reducing the risk of acquiring HIV. PrEP is underused due, in part, to prescriber inaccessibility. The overall aim of this study was to evaluate the impact of pharmacist PrEP management (including prescribing and monitoring) on clinical and acceptance outcomes in patients who are at high risk for HIV exposure.

**Methods:**

Pharmacist-led PrEP management was guided by a prescribing protocol and implemented at 10 community pharmacies in Nova Scotia over 6 months. Baseline and follow-up bloodwork determined HIV status, coinfection(s), and other eligibility criteria for initial and refill PrEP prescriptions. Patient acceptance was measured according to the theoretical framework for acceptability of health care interventions.

**Results:**

Forty-five participants met eligibility criteria, and 37 remained for the study duration. Around half of the participants had never used PrEP before, and all identified as men who have sex with men or transgender women. Participants were highly accepting of the service and agreed that pharmacist-led PrEP management should always be available. Few reported privacy or stigma/discrimination concerns. All participants remained HIV-negative during study participation, and participants with coinfections were linked with care (*n* = 4).

**Interpretation:**

The service was acceptable and effective for patients. Future work is required to reach underserved populations, particularly individuals with injection-related HIV risk factors.

**Conclusions:**

Pharmacist-led PrEP management can provide an alternative way to obtain PrEP for higher-risk patients. This study resulted in a regulation change on July 1, 2024 that authorized pharmacists to prescribe PrEP in Nova Scotia.

**Knowledge into Practice**

- Human immunodeficiency virus (HIV) pre-exposure prophylaxis (PrEP) is a highly effective strategy to reduce the incidence of HIV, but access to HIV PrEP prescribing limits its use.
- Community pharmacists in Nova Scotia effectively assessed patient eligibility, and prescribed and monitored HIV PrEP. Patients accessing this service found it accessible and were satisfied with the care they received.
- HIV PrEP prescribing is feasible, acceptable, and well-positioned within the community pharmacy setting.

## Introduction

Pre-exposure prophylaxis (PrEP) with tenofovir disoproxil fumarate/emtricitabine for the prevention of human immunodeficiency virus (HIV) infection is a highly effective tool to reduce the incidence of HIV.^1^ PrEP is associated with up to a 99% relative reduction in HIV incidence from sexual exposure in gay and bisexual men who have sex with men (gbMSM),^2,3^ up to 95% for heterosexual exposure,^4^ and up to 84% for injection drug use when taken once daily with high levels of adherence.^4,5^ It is estimated that for every 13 high-risk individuals treated with daily PrEP, at least 1 new HIV infection can be prevented.^2^ Routine care for PrEP prescribing requires regular sexually transmitted infection (STI) testing, creating additional opportunities for STI detection and treatment, as well as general health promotion. PrEP is recommended for populations at greater risk of HIV transmission, including gbMSM and transgender women, persons who inject drugs, those who exchange sex for money or drugs, or HIV-negative people in sexual relationships with known HIV-positive partners.^6^

Despite being highly effective in preventing HIV transmission, barriers prevent populations at greater risk of HIV from accessing PrEP, leading to significant underuse.^4,7,8^ People receiving PrEP need regular and consistent contact with practitioners for testing and prescribing, which can be limited by the availability of primary care providers and sexual health clinics. Therefore, many potential PrEP users cite prescriber access as a major barrier to receiving therapy.^9^ Long wait times for specialty clinics, lack of knowledge about PrEP use and laboratory monitoring requirements by some prescribers, and experiences of past stigma or discrimination within primary care settings were also reported as major barriers to accessing PrEP.^4^

These barriers, combined with the fact that over 150,000 individuals in Nova Scotia do not have access to a family physician or a nurse practitioner, require creative solutions.^10^ PrEP management by pharmacists may offer a solution to overcome these barriers and improve PrEP access to eligible populations. Collaborative practice models exist for pharmacist PrEP prescribing but fully recognize the service within pharmacists’ scope of practice is relatively new.^11-13^ Recent changes in the United States allow some pharmacists in certain states to prescribe PrEP outside of collaborative practice agreements with other authorized prescribers.^13^ At the time of this study, most pharmacists in Canada are not authorized to prescribe PrEP without the implementation of a collaborative practice framework. Although pharmacists in Alberta technically have authority, they are not included on the designated HIV PrEP prescriber list that is required for patients to receive coverage.

**Knowledge into Practice**

- La prophylaxie pré-exposition (PrEP) au VIH est une stratégie très efficace pour réduire l’incidence du VIH, mais l’accès à la prescription de la PrEP pour le VIH limite son utilisation.
- Les pharmaciens communautaires de la Nouvelle-Écosse ont évalué efficacement l’admissibilité des patients, et ont prescrit et contrôlé la PrEP pour le VIH. Les patients qui ont accédé à ce service l’ont trouvé accessible et ont été satisfaits des soins qu’ils ont reçus.
- La prescription de la PrEP pour le VIH est faisable, acceptable et bien positionnée dans le milieu de la pharmacie communautaire.

The overall aim of this study was to evaluate the impact of pharmacist PrEP management (including prescribing and monitoring) on outcomes in patients who are at high risk for HIV in Nova Scotia. Specific objectives were the following:

1. To determine patients’ acceptance of implementing PrEP prescribing by pharmacists across the 7 constructs of the theoretical framework of acceptability of health care interventions (TFA).^14^
2. To determine the rates of clinical outcomes (HIV positivity rates, identification of coinfections, and other laboratory abnormalities) when PrEP is prescribed by pharmacists.

## Methods

### Study design and setting

This was a 6-month implementation and evaluation study of an independent PrEP prescribing service by pharmacists in the Halifax Regional Municipality of Nova Scotia that occurred from March 2023 through October 2023. This study is reported using the Standards for Reporting Implementation Studies (STaRI) and is included in Appendix 1, available online under Supplementary Materials.^15^

The Halifax Regional Municipality is an urban centre with a population greater than 400,000 people.^16^ The 10 community pharmacy sites selected to participate through an application process had access to provincial software for viewing laboratory results and at least 2 pharmacists on staff for continuity of care. For the purposes of the study, the provincial pharmacy regulatory body (Nova Scotia College of Pharmacists [NSCP]) approved PrEP prescribing under its *Standards of Practice: Prescribing Drugs—Appendix I: Prescribing in Accordance with an Approved Research or Pilot Protocol*. The prescribing protocol was developed by clinical pharmacists based on Canadian PrEP prescribing guidelines^6^ and was reviewed and approved by 3 physicians (including 2 infectious disease specialists and 1 primary care physician). At the time of study launch, not all pharmacists were able to order laboratory tests. To address this, a collaborative practice framework was initiated between pharmacists and a study physician. This allowed the results from preauthorized laboratory requisitions to be provided to study participants by study pharmacists for the purpose of assessing participant eligibility and monitoring while on PrEP and linkage to care for abnormal results.

### Research ethics

This project was approved by the Dalhousie University Health Sciences Research Ethics Board (file # 2022-6044) on November 3, 2022. All participants provided written informed consent before participating and for anonymized patient information to be published.

### Sample size

Based on the implementation nature of this study and the funding available to cover drug costs for enrolled patients, a maximum sample size of 50 participants was determined a priori. This also aligned with previous studies that reported on patient-reported outcomes pertaining to PrEP prescribing by pharmacists.^12,13^

### Participants

Nova Scotia residents were eligible to participate if they identified with a group at high risk for HIV transmission.^5^ This included gbMSM or transgender women who had a previous bacterial STI or syphilis, used non-occupational-related HIV postexposure prophylaxis at least twice, were in a sexual relationship with a known HIV-positive partner, or scored 11 points or higher on the HIV Incidence Risk Index for Men who have Sex with Men (HIRI-MSM).^17^ Persons who inject drugs or heterosexual individuals in sexual relationships with a known HIV-positive partner were also eligible. Participant recruitment occurred via online news media through Dalhousie University and social media (X, Instagram) from researchers, collaborators, and pharmacy accounts, with advertisements shared widely. The principal investigator also appeared on 2 television interviews for study promotion.

### Procedures

Interested participants contacted the study team via email or phone and were initially screened by the principal investigator via phone to review study procedures, ensure the participant identified with 1 of the eligible populations above, and obtain study consent. Screening occurred via a standardized script, checklist, and form. The name and contact information of those meeting these criteria were then given to a participating pharmacist of the participants’ choice for study procedure initiation. The Pharmacy Association of Nova Scotia recruited pharmacy sites on an application basis and were not limited funder sites. Participants completed 4 in-person appointments throughout the study. Pharmacists reviewed participant eligibility for PrEP according to the approved protocol (Appendix 2, available online under Supplementary Materials) at appointment 1, including medical history, concurrent medication, and sexual or injection drug use history. If participants were eligible for PrEP, a laboratory requisition was provided for HIV and coinfection (chlamydia, gonorrhea, syphilis, hepatitis B and C), along with baseline laboratory testing to ensure appropriateness of once-daily oral tenofovir disoproxil fumarate/emtricitabine. Study pharmacists assisted participants in booking appointments for blood collection from Nova Scotia Health laboratories that reported results available for viewing by pharmacists. Participants were provided with information to access swab-based gonorrhea and chlamydia testing from sexual health and sexually transmitted infection clinics in Halifax. Those not deemed eligible for PrEP were not provided with laboratory requisitions, ceased all study procedures, and were invited to complete the voluntary survey. Upon receipt of the laboratory results and PrEP eligibility confirmation, pharmacists provided participants with a 30-day PrEP supply and a laboratory requisition for HIV testing (appointment 2). Pharmacists also provided education and counselling for PrEP according to Canadian guidelines. If the participant remained eligible upon assessment of continuing risk and laboratory results at appointment 3, a 60-day PrEP supply and laboratory requisition for bloodwork to be completed before the next refill were provided. Participants received a final 90-day PrEP supply at appointment 4 if they remained eligible according to the protocol. Medication costs and dispensing fees were covered by the study if patients did not have 100% coverage through other sources (private or public insurance). Pharmacies received clinical service fees from the study in consultation with the Pharmacy Association of Nova Scotia ($75 for appointment 1, $20 for each additional appointment). For the purposes of this study, only tenofovir disoproxil fumarate/emtricitabine was used as the PrEP formulation based on cost, coverage, and availability in Nova Scotia.

### Data collection

Pharmacists collected participants’ data (demographics, eligibility information, laboratory results) online using REDCap electronic data capture tools.^18,19^ Participants were asked to complete an anonymous and voluntary 5- to 10-minute electronic questionnaire developed according to the TFA to assess their acceptance and satisfaction with service delivery after each appointment. The TFA for health care interventions consists of 7 constructs of acceptability and can be used to examine health care users’ or providers’ cognitive and emotional responses to an intervention.^14^

The questionnaire consisted of demographic questions, 16 Likert-type questions, and an open-ended question for any other feedback not captured by the other questionnaire items.

### Data analysis

Participant demographics, eligibility information, and laboratory results were analyzed descriptively. Participants’ acceptance and satisfaction results were analyzed by appointment using descriptive statistics.

## Results

Fifty participants were screened, and 45 met enrollment criteria and were included in this analysis. The 5 not meeting criteria did not meet 1 of the risk categories as designated in the protocol. Participant characteristics are reported in Table 1. Many characteristics were collected through the voluntary participant survey, and therefore, some characteristics are not available for all participants. Most respondents were between 25 and 45 years of age (75.7%), identified as male (93.9%) and sexually diverse (not heterosexual) (92.1%), and reported their cultural background as White/European (66.7%). Approximately half of the participants had never used PrEP before study enrollment (51.5%), while the other half were current (24.2%) or former (24.2%) PrEP users at the first study eligibility appointment. All participants met PrEP eligibility based on risk factors reported as a HIRI-MSM score greater than 11 (78.2%), a previous diagnosis with syphilis or a rectal sexually transmitted infection (16.4%), an ongoing relationship with an HIV-positive partner (3.6%), or recurrent use of HIV postexposure prophylaxis (1.8%) (Table 2).

**Table 1:** Participant sociodemographic characteristics

	*n* (%)	Total
Age		37
18-25	5 (13.5)	
25-35	19 (51.4)	
35-45	9 (24.3)	
45-55	3 (8.1)	
55+	1 (2.7)	
Identity		38
Sexually diverse	35 (92.1)	
Gender diverse	2 (5.3)	
A person who injects drugs	0	
None of the above	1 (2.6)	
Gender		33
Male	28 (84.8)	
Cisgender male	3 (9.1)	
Non-binary	1 (3.0)	
Male non-conforming	1 (3.0)	
Cultural background		45
African Nova Scotian	2 (4.4)	
Black	1 (2.2)	
European/White	30 (66.7)	
East Asian	1 (2.2)	
South Asian	2 (4.4)	
Southeast Asian	1 (2.2)	
First Nations or Indigenous	2 (4.4)	
Hispanic or Latinx	3 (6.7)	
Middle Eastern	1 (2.2)	
Other	1 (2.2)	
Prefer not to answer	1 (2.2)	
Insurance coverage		39
Private	23 (59.0)	
Public	1 (2.6)	
No coverage	15 (38.5)	
History of PrEP use		33
Current PrEP user	8 (24.2)	
Never PrEP user	17 (51.5)	
Former PrEP user	8 (24.2)	

PrEP, pre-exposure prophylaxis.

**Table 2:** PrEP eligibility and risk factors

	*n* (%)	Total
PrEP eligibility		45
Men who have sex with men/ transgender women	45 (100)	
Heterosexual with HIV+ partner	0	
Injection drug use	0	
Risk factors		55
Previous syphilis or rectal sexually transmitted infection	9 (16.4)	
Recurrent use of HIV postexposure prophylaxis	1 (1.8)	
Sexual relationship with HIV+ partner	2 (3.6)	
HIV Incidence Risk Index for MSM >11	43 (78.2)	

HIV, human immunodeficiency virus; MSM, men who have sex with men; PrEP, pre-exposure prophylaxis.

All participants (*n* = 42) who completed bloodwork were HIV-negative at the initial eligibility visit and remained HIV-negative while participating in the study (Table 3). Half of the participants were found to have hepatitis A immunity (51.2%) and three-quarters to have hepatitis B immunity (75.6%). Pharmacists offered vaccination to eligible participants according to Nova Scotia’s publicly funded eligibility policy recommendations. At baseline, pharmacists identified 1 case each of gonorrhea, syphilis, and active hepatitis B in different patients. At the second follow-up appointment, 1 participant was found to have chlamydia. The participants with gonorrhea, syphilis, and chlamydia were referred to the study physician for treatment, and their participation in the study continued. The case with active hepatitis B was withdrawn from the study and referred for specialized care.

**Table 3:** Laboratory results from the prescribing and refill appointments

	Prescribing appointment, *n* (%) *n* = 41*	Refill appointment 1, *n* (%) *n* = 37*	Refill appointment 2, *n* (%) *n* = 35*
HIV status			
Positive	0	0	0
Negative	41 (100)	37 (100)	35 (100)
HIV viral load			
Undetectable	20 (48.8)	26 (70.3)	25 (71.4)
Detectable	0	0	0
Not reported/ unavailable	21 (51.2)	11 (29.7)	10 (28.6)
Hepatitis A immunity			
Immune	21 (51.2)	—	—
Not immune	20 (48.8)	—	—
Hepatitis B immunity			
Immune	31 (75.6)	—	—
Not immune	10 (24.4)	—	—
Hepatitis B infection			
Positive	1 (2.4)	—	—
Negative	40 (97.6)	—	—
Hepatitis C status			
Positive	0	—	—
Negative	41 (100)	—	—
Complete blood count			
Within normal limits	38 (92.7)	—	—
Abnormal	3 (7.3)	—	—
Urinalysis			
Within normal limits	35 (85.4)	—	—
Abnormal	6 (14.6)	—	—
Estimated glomerular filtration rate			
<60 mL/min/1.73 m²	0	0	0
>60 mL/min/1.73 m²	41 (100)	37 (100)	35 (100)
β-Human chorionic gonadotropin			
Positive	0	0	0
Negative	1 (2.4)	1 (2.7)	1 (2.9)
Not applicable/not assessed	40 (97.6)	36 (97.3)	34 (97.1)
Alanine transaminase			
Within normal limits	39 (95.1)	35 (94.6)	33 (94.3)
>54 U/L (male) or >44 U/L (female)	2 (4.9)	2 (5.4)	2 (5.7)
Coinfections			
No coinfections	38 (92.7)	—	32 (91.4)
Coinfections	3 (7.3)	—	3 (8.6)
* Syphilis*	1^†^	—	0^‡^
* Chlamydia*	0	—	1
* Gonorrhea*	1	—	0

* In total, 42, 39, and 37 participants attended the prescribing, refill 1, and 2 appointments, respectively, but not all laboratory results were provided to the research team. All participants remained HIV-negative while enrolled in the study, and participant laboratory results not reflected in this table were within normal limits or otherwise clinically unremarkable, as confirmed by the treating pharmacist and study physician.

† One participant had a history of syphilis; therefore, immunoglobulin G/immunoglobulin M (IgG/IgM) was persistently positive.

‡ Two participants had a history of syphilis; therefore, IgG/IgM was persistently positive.

All screened participants attended appointment 1 (eligibility), 42 (93.3%) attended appointment 2 (first prescribing appointment), and 39 (86.7%) and 37 (82.2%) attended appointments 3 and 4, respectively. Of the 8 of 45 participants who did not attend all appointments, 1 was withdrawn from the study following the discovery of hepatitis B infection, another was withdrawn after experiencing intolerable adverse effects, 1 was unable to complete the eligibility bloodwork, and 5 had relocated or were lost to follow-up.

Participants were highly accepting of PrEP pharmacist prescribing across all TFA constructs, with Likert-style questionnaire responses represented in Table 4 for appointments 1 and 2. Likert-style question responses from appointments 3 and 4 can be found in Appendix 3, available online under Supplementary Materials. Greater than 95% of participants reported feeling comfortable seeing the pharmacist, would recommend seeing a pharmacist for PrEP to a friend, and agreed PrEP prescribing should always be available in pharmacies. The majority found the pharmacy accessible. Most reported they did not face stigma or discrimination at the pharmacy, and most felt it is important for the pharmacy to have a private counselling room. Very few participants (3%) worried about privacy, and most participants did not feel concerned about privacy at the pharmacy. Most participants felt it was important that PrEP prescribing at the pharmacy is free of charge, that health questions and test results are kept private and confidential, and that PrEP prescribing through pharmacies helps their community. The majority felt confident they understood the process of obtaining PrEP from the pharmacist. Most did not feel pharmacist PrEP prescribing would have negative consequences for them; however, approximately half of the respondents would not have been willing to pay for PrEP out-of-pocket. Participants felt confident that the pharmacist did a good job and felt capable of communicating their needs to the pharmacist. Participants felt confident in their pharmacist as a PrEP prescriber but also noted that it is important for the service to be free to them.

**Table 4:** Participant questionnaire responses to Likert-type questions categorized by theoretical framework of acceptability for health care interventions construct after the eligibility appointment (appointment 1) and the first prescribing appointment (appointment 2)

		Agree, *n* (%)	Neutral, *n* (%)	Disagree, *n* (%)	Prefer not to answer, *n* (%)
Effective attitude					
I felt comfortable seeing the pharmacist about PrEP today.	Eligibility	36 (97.3)	0	1 (3.2)	1
	Prescribing	30 (96.8)	0	1 (3.2)	0
I would recommend to my friends that they see a pharmacist for PrEP.	Eligibility	37 (100)	0	0	1
	Prescribing	30 (96.8)	0	1 (3.2)	0
I believe PrEP prescribing should always be available in pharmacies.	Eligibility	37 (100)	0	0	1
	Prescribing	30 (96.8)	0	1 (3.2)	0
Burden					
I faced stigma or discrimination when I came to the pharmacy.	Eligibility	0	1 (2.7)	36 (97.3)	1
	Prescribing	3 (9.7)	0	28 (90.3)	0
The pharmacy is accessible to me.	Eligibility	36 (97.3)	1 (2.7)	0	1
	Prescribing	30 (96.8)	0	1 (3.2)	0
Having a private room for consultation is important.	Eligibility	32 (86.5)	4 (10.8)	1 (2.7)	1
	Prescribing	26 (83.9)	3 (9.7)	2 (6.5)	0
Ethicality					
It is important that PrEP prescribing at the pharmacy is free.	Eligibility	35 (97.2)	1 (2.8)	0	2
	Prescribing	30 (96.8)	0	1 (3.2)	0
It is important that my health questions and test results are kept private and confidential.	Eligibility	34 (94.4)	2 (5.6)	0	2
	Prescribing	29 (93.5)	1 (3.2)	1 (3.2)	0
Having PrEP prescribing available through pharmacies will help my community.	Eligibility	36 (100)	0	0	2
	Prescribing	30 (96.8)	0	1 (3.2)	0
Intervention coherence					
I am confident that I understand the process of obtaining PrEP from the pharmacist.	Eligibility	37 (100)	0	0	1
	Prescribing	30 (96.8)	0	1 (3.2)	0
PrEP prescribing by a pharmacist will damage my relationship with my other health care providers.	Eligibility	2 (5.4)	1 (2.7)	34 (91.9)	1
	Prescribing	3 (9.7)	1 (3.2)	27 (87.1)	0
Opportunity costs					
I would be willing to pay for PrEP if it wasn’t covered by the study (approx. $250 per month).	Eligibility	9 (24.3)	7 (18.9)	21 (56.8)	1
	Prescribing	11 (35.5)	6 (19.4)	14 (45.2)	0
Pharmacist prescribing of PrEP will have negative consequences for me.	Eligibility	1 (2.7)	1 (2.7)	35 (94.6)	1
	Prescribing	1 (3.2)	0	30 (96.8)	0
I am worried about the privacy offered by the pharmacy when discussing PrEP with the pharmacist.	Eligibility	1 (2.7)	2 (5.4)	34 (91.9)	1
	Prescribing	1 (3.2)	2 (6.5)	28 (90.3)	0
Perceived effectiveness					
I am confident that the pharmacist did a good job today.	Eligibility	37 (100)	0	0	1
	Prescribing	30 (96.8)	0	1 (3.2)	0
Self-efficacy					
I am capable of telling a pharmacist about my needs for PrEP.	Eligibility	37 (100)	0	0	1
	Prescribing	30 (96.8)	0	1 (3.2)	0

PrEP, pre-exposure prophylaxis.

## Discussion

This study implemented and evaluated pharmacist-led PrEP management in Nova Scotia. Pharmacists were able to prescribe PrEP, identify coinfections and other laboratory abnormalities, and link patients to further care when needed. PrEP prescribing by pharmacists is acceptable to patients across all domains of the TFA. These results contribute to the existing literature by demonstrating the acceptability of pharmacist PrEP prescribing and offer further insight into the considerations of broader implementation (e.g., laboratory abnormality assessments, provision of vaccines, and linkage to care).

The key finding of this study was the acceptance of pharmacist PrEP prescribing by patients. Eighty-two percent of participants were retained through appointment 4. Study participants were positive across all domains of the TFA and spoke highly of the perceived effectiveness of the service and their preference to have PrEP prescribed by pharmacists, as compared to other health care professionals. This may be explained by the fact that pharmacists are accessible health care providers, simplification of the process of obtaining PrEP with fewer contact points with the health care system, and the availability of a consistent prescriber, allowing continuity of care when participants lacked a primary care provider. This model demonstrated the ability to reach individuals who had never used PrEP before and a sexually diverse population. These findings align with preliminary studies conducted by the research team that found high perceived acceptability by target PrEP users before service implementation and with other implementation studies through collaborative practice agreements in other settings.^20,21^ Notably, about half of the participants would not be willing to pay for PrEP, suggesting that measures beyond task-shifting PrEP management to pharmacists are required to facilitate PrEP use by those who may benefit from it. This is likely especially true for individuals with neither public nor private insurance (38.5% in this sample). Disruptions in relationships with other health care providers were not of concern, and participants commented on their confidence that the pharmacist could appropriately link them with further care. This is an important finding as previous studies have reported linkage to care to be a barrier for pharmacist-led PrEP prescribing.^22,23^

A finding that differed from existing literature was the lack of privacy concerns identified by participants in this study. Previous studies, including preliminary work by this research team, have reported that privacy is a major barrier to providing confidential sexual health services and may be a reason for poor uptake or initiation of collaborative practice agreements.^21,23,24^ While the participants in this implementation study did not appear to be concerned about privacy, it should be noted that all participating pharmacies used private counselling rooms for study procedures. The pharmacies were also all located in urban areas, where privacy may be less of a concern compared to rural locations.

The findings from this study were used by the NSCP to broadly authorize pharmacists in Nova Scotia to prescribe PrEP as of July 1, 2024. This demonstrates the importance of well-designed and collaborative research-informed approaches in advancing pharmacy practice change. Key facilitators for the success of the study and scope authorization included close collaborations with the provincial regulator, pharmacy association, physicians, community organizations, and researchers on all aspects of study design, implementation, and evaluation. The ability to study a new scope of practice through an approved research protocol was also foundational to determining real-world facilitators and barriers to broader adoption.

### Limitations

First, the sample size was limited to 50 patients, and there was attrition throughout the study. While the sample size was dependent on available resources, the attrition rate was not unexpected as PrEP is a preventative medication, and changes in participants’ relationship status or sexual practices may have altered their decision to remain enrolled. Second, although PrEP prescribing was deemed part of pharmacists’ scope of practice for the purposes of this study by the regulatory authority, a collaborative practice agreement was necessary to order and view the results of laboratory tests for pharmacists who lacked access to view the results during the study period. This may have underestimated the burden of follow-up on laboratory abnormalities as the study physician was directly involved in communicating results to participants. As laboratory test ordering continues to expand and PrEP prescribing is now within scope, additional research should be conducted to determine competency when addressing these abnormalities as part of routine care. Finally, participants were primarily urban, and all met eligibility criteria as gbMSM or transgender women. Results of the study are therefore unable to be applied to other populations typically indicated for PrEP, including persons who inject drugs or cisgendered, heterosexual individuals, and future work focused on improving access for all key populations is warranted. This potential selection bias likely occurred because study promotion activities were largely limited to public and social network advertisements shared by investigators with close ties to the gbMSM community.

## Conclusion

Pharmacist prescribing for PrEP was successfully implemented in community pharmacies in Nova Scotia. This study found PrEP prescribing by pharmacists was feasible, effective, and acceptable to patients. These findings support pharmacist prescribing of PrEP as part of their scope of practice and may improve access to sexual health services and increase linkage to care for at-risk populations. Future studies should be conducted to determine the overall impact of PrEP prescribing by pharmacists on patients, the pharmacy workforce, and the health care system. ■

## Supplemental Material

sj-pdf-1-cph-10.1177_17151635251355277 – Supplemental material for Implementation and acceptance of pharmacists’ prescribing of human immunodeficiency virus (HIV) pre-exposure prophylaxis (PrEP) Supplemental material, sj-pdf-1-cph-10.1177_17151635251355277 for Implementation and acceptance of pharmacists’ prescribing of human immunodeficiency virus (HIV) pre-exposure prophylaxis (PrEP) by Mackenzie d’Entremont-Harris, Tasha Diana Ramsey, Kathleen MacNabb, Andrea Murphy, Andrea Bishop, Jennifer E. Isenor, Deborah V. Kelly, Yazid N. Al Hamarneh, Matthew Lee, Abbey Ferguson, Kirk Furlotte, Lisa Woodill, Todd Hatchette and Kyle John Wilby in Canadian Pharmacists Journal / Revue des Pharmaciens du Canada

sj-pdf-2-cph-10.1177_17151635251355277 – Supplemental material for Implementation and acceptance of pharmacists’ prescribing of human immunodeficiency virus (HIV) pre-exposure prophylaxis (PrEP) Supplemental material, sj-pdf-2-cph-10.1177_17151635251355277 for Implementation and acceptance of pharmacists’ prescribing of human immunodeficiency virus (HIV) pre-exposure prophylaxis (PrEP) by Mackenzie d’Entremont-Harris, Tasha Diana Ramsey, Kathleen MacNabb, Andrea Murphy, Andrea Bishop, Jennifer E. Isenor, Deborah V. Kelly, Yazid N. Al Hamarneh, Matthew Lee, Abbey Ferguson, Kirk Furlotte, Lisa Woodill, Todd Hatchette and Kyle John Wilby in Canadian Pharmacists Journal / Revue des Pharmaciens du Canada

sj-pdf-3-cph-10.1177_17151635251355277 – Supplemental material for Implementation and acceptance of pharmacists’ prescribing of human immunodeficiency virus (HIV) pre-exposure prophylaxis (PrEP) Supplemental material, sj-pdf-3-cph-10.1177_17151635251355277 for Implementation and acceptance of pharmacists’ prescribing of human immunodeficiency virus (HIV) pre-exposure prophylaxis (PrEP) by Mackenzie d’Entremont-Harris, Tasha Diana Ramsey, Kathleen MacNabb, Andrea Murphy, Andrea Bishop, Jennifer E. Isenor, Deborah V. Kelly, Yazid N. Al Hamarneh, Matthew Lee, Abbey Ferguson, Kirk Furlotte, Lisa Woodill, Todd Hatchette and Kyle John Wilby in Canadian Pharmacists Journal / Revue des Pharmaciens du Canada
