# Supplementary material for: Implementation and acceptance of pharmacists’ prescribing of human immunodeficiency virus (HIV) pre-exposure prophylaxis (PrEP)
Source: Can Pharm J (Ott). 2025 Aug 22;158(5):302–11. doi: 10.1177/17151635251355277 (PMC12373644; doi:10.1177/17151635251355277)
Supplement: sj-pdf-1-cph-10.1177_17151635251355277 – Supplemental material for Implementation and acceptance of pharmacists’ prescribing of human immunodeficiency virus (HIV) pre-exposure prophylaxis (PrEP) [file sj-pdf-1-cph-10.1177_17151635251355277.pdf]

Appendix 1: Standards for reporting implementation studies: The StaRI checklist

Checklist item		Reported on page #	Implementation strategy	Reported on page #	Intervention
			"Implementation strategy" refers to how the intervention was implemented.		"Intervention" refers to the health care or public health intervention that is being implemented.
**Title and abstract**					
Title	**1**	1	Identification as an implementation study, and description of the methodology in the title and/or keywords.		
Abstract	**2**	1	Identification as an implementation study, including a description of the implementation strategy to be tested, the evidence-based intervention being implemented, and defining the key implementation and health outcomes.		
**Introduction**					
Introduction	**3**	3	Description of the problem, challenge, or deficiency in health care or public health that the intervention being implemented aims to address.		
Rationale	**4**	3,4	The scientific background and rationale for the implementation strategy (including any underpinning theory/framework/model, how it is expected to achieve its effects, and any pilot work).	3,4	The scientific background and rationale for the intervention being implemented (including evidence about its effectiveness and how it is expected to achieve its effects).
Aims and objectives	**5**	4	The aims of the study, differentiating between implementation objectives and any intervention objectives.		
**Methods: Description**					
Design	**6**	4,5	The design and key features of the evaluation (cross-referencing to any appropriate methodology reporting standards) and any changes to study protocol, with reasons.		
Context	**7**	4	The context in which the intervention was implemented. (Consider social, economic, policy, health care, organizational barriers, and facilitators that might influence implementation elsewhere).		
Targeted "sites"	**8**	4	The characteristics of the targeted "site(s)" (e.g., locations/personnel/resources, etc.) for implementation and any eligibility criteria.	5	The population targeted by the intervention and any eligibility criteria.
Description	**9**	5.6	A description of the implementation strategy.	5.6	A description of the intervention.
Subgroups	**10**	N/A	Any subgroups recruited for additional research tasks, and/or nested studies are described.		

**Methods: Evaluation**					
Outcomes	**11**	4	Defined prespecified primary and other outcome(s) of the implementation strategy, and how they were assessed. Document any predetermined targets.	4	Defined prespecified primary and other outcome(s) of the intervention (if assessed), and how they were assessed. Document any predetermined targets.
Process evaluation	**12**	5,6	Process evaluation objectives and outcomes related to the mechanism by which the strategy is expected to work.		
Economic evaluation	**13**	N/A	Methods for resource use, costs, economic outcomes, and analysis for the implementation strategy.	N/A	Methods for resource use, costs, economic outcomes, and analysis for the intervention.
Sample size	**14**	5	Rationale for sample sizes (including sample size calculations, budgetary constraints, practical considerations, data saturation, as appropriate).		
Analysis	**15**	5,6	Methods of analysis (with reasons for that choice).		
Subgroup analyses	**16**	N/A	Any a priori subgroup analyses (e.g., between different sites in a multicentre study, different clinical or demographic populations) and subgroups recruited to specific nested research tasks.		
**Results**					
Characteristics	**17**	7	Proportion recruited and characteristics of the recipient population for the implementation strategy.	7	Proportion recruited and characteristics (if appropriate) of the recipient population for the intervention.
Outcomes	**18**	7,8	Primary and other outcome(s) of the implementation strategy.	7,8	Primary and other outcome(s) of the intervention (if assessed).
Process outcomes	**19**	N/A	Process data related to the implementation strategy mapped to the mechanism by which the strategy is expected to work.		
Economic evaluation	**20**	N/A	Resource use, costs, economic outcomes, and analysis for the implementation strategy.	N/A	Resource use, costs, economic outcomes, and analysis for the intervention.
Subgroup analyses	**21**	N/A	Representativeness and outcomes of subgroups including those recruited to specific research tasks.		
Fidelity/ adaptation	**22**	N/A	Fidelity to implementation strategy as planned and adaptation to suit context and preferences.		Fidelity to delivering the core components of intervention (where measured).
Contextual changes	**23**	10	Contextual changes (if any) which may have affected outcomes.		

Harms	**24**	N/A	All important harms or unintended effects in each group.		
**Discussion**					
Structured discussion	**25**	8,9	Summary of findings, strengths, and limitations; comparisons with other studies, conclusions, and implications.		
Implications	**26**	11	Discussion of policy, practice, and/or research implications of the implementation strategy (specifically including scalability).	11	Discussion of policy, practice, and/or research implications of the intervention (specifically including sustainability).
**General**					
Statements	**27**	5, 11	Include statement(s) on regulatory approvals (including, as appropriate, ethical approval, confidential use of routine data, governance approval), trial/study registration (availability of protocol), funding, and conflicts of interest.		

d'Entremont-Harris M, Ramsey TD, MacNabb K, et al. Implementation and acceptance of pharmacists' prescribing of Human Immunodeficiency Virus (HIV) Pre-Exposure Prophylaxis (PrEP). *Can Pharm J (Ott)* 2025;158. DOI 10.1177/17151635231355277.
