# Supplementary material for: Implementation and acceptance of pharmacists’ prescribing of human immunodeficiency virus (HIV) pre-exposure prophylaxis (PrEP)
Source: Can Pharm J (Ott). 2025 Aug 22;158(5):302–11. doi: 10.1177/17151635251355277 (PMC12373644; doi:10.1177/17151635251355277)
Supplement: sj-pdf-2-cph-10.1177_17151635251355277 – Supplemental material for Implementation and acceptance of pharmacists’ prescribing of human immunodeficiency virus (HIV) pre-exposure prophylaxis (PrEP) [file sj-pdf-2-cph-10.1177_17151635251355277.pdf]

Appendix 3: Participant questionnaire responses to Likert-item type questions categorized by Theoretical Framework of Acceptability for healthcare interventions construct after the first (Appointment 3) and second (Appointment 4) refill appointments.

		Agree	Neutral	Disagree	Prefer not to answer
**Affective attitude**		*n* (%)			
I felt comfortable seeing the pharmacist about PrEP today.	REFILL 1	27 (100)	0	0	0
	REFILL 2	24 (100)	0	0	0
I would recommend to my friends that they see a pharmacist for PrEP.	REFILL 1	27 (100)	0	0	0
	REFILL 2	24 (100)	0	0	0
I believe PrEP prescribing should always be available in pharmacies.	REFILL 1	27 (100)	0	0	0
	REFILL 2	24 (100)	0	0	0
**Burden**					
I faced stigma or discrimination when I came to the pharmacy.	REFILL 1	1 (3.7)	0	26 (96.3)	0
	REFILL 2	0	0	24 (100)	0
The pharmacy is accessible to me.	REFILL 1	26 (96.3)	1 (3.7)	0	0
	REFILL 2	24 (100)	0	0	0
Having a private room for consultation is important.	REFILL 1	24 (88.9)	2 (7.4)	1 (3.7)	0
	REFILL 2	20 (83.3)	3 (12.5)	1 (4.2)	0
**Ethicality**					
It is important that PrEP prescribing at the pharmacy is free.	REFILL 1	26 (96.3)	1 (3.7)	0	0
	REFILL 2	22 (91.7)	2 (8.3)	0	0
It is important that my health questions and test results are kept private and confidential.	REFILL 1	26 (96.3)	1 (3.7)	0	0
	REFILL 2	23 (95.8)	1 (4.2)	0	0
Having PrEP prescribing available through pharmacies will help my community.	REFILL 1	27 (100)	0	0	0
	REFILL 2	24 (100)	0	0	0
**Intervention coherence**					
I am confident that I understand the process of obtaining PrEP from the pharmacist.	REFILL 1	27 (100)	0	0	0
	REFILL 2	24 (100)	0	0	0
PrEP prescribing by a pharmacist will damage my relationship with my other healthcare providers.	REFILL 1	2 (7.4)	0	25 (92.6)	0
	REFILL 2	0	0	24 (100)	0

**Opportunity costs**					
I would be willing to pay for PrEP if it wasn't covered by the study (appox. $250 per month).	REFILL 1	6 (22.2)	7 (25.9)	14 (51.9)	0
	REFILL 2	7 (29.2)	3 (12.5)	14 (58.3)	0
Pharmacist prescribing of PrEP will have negative consequences for me.	REFILL 1	4 (14.8)	0	23 (85.2)	0
	REFILL 2	0	0	24 (100)	0
I am worried about the privacy offered by the pharmacy when discussing PrEP with the pharmacist.	REFILL 1	1 (3.7)	0	26 (96.3)	0
	REFILL 2	0	1 (4.2)	23 (95.8)	0
**Perceived effectiveness**					
I am confident that the pharmacist did a good job today.	REFILL 1	27 (100)	0	0	0
	REFILL 2	24 (100)	0	0	0
**Self-efficacy**					
I am capable of telling a pharmacist about my needs for PrEP.	REFILL 1	27 (100)	0	0	0
	REFILL 2	24 (100)	0	0	0

d'Entremont-Harris M, Ramsey TD, MacNabb K, et al. Implementation and acceptance of pharmacists' prescribing of Human Immunodeficiency Virus (HIV) Pre-Exposure Prophylaxis (PrEP). Can Pharm J (Ott) 2025;158. DOI 10.1177/17151635231355277.
