# Supplementary material for: Implementation and acceptance of pharmacists’ prescribing of human immunodeficiency virus (HIV) pre-exposure prophylaxis (PrEP)
Source: Can Pharm J (Ott). 2025 Aug 22;158(5):302–11. doi: 10.1177/17151635251355277 (PMC12373644; doi:10.1177/17151635251355277)
Supplement: sj-pdf-3-cph-10.1177_17151635251355277 – Supplemental material for Implementation and acceptance of pharmacists’ prescribing of human immunodeficiency virus (HIV) pre-exposure prophylaxis (PrEP) [file sj-pdf-3-cph-10.1177_17151635251355277.pdf]

Appendix 2
Pre-exposure prophylaxis (PrEP) for HIV prescribing protocol

Patient information

Name: ______________________________ Preferred name/alias: ________________________

HCN: _______________________________ Phone number: ______________________________

Street address: ____________________ City/town: ____________________________

Province: _________________________ Postal code: ____________

Date of birth: _______________________ Age: ____

Gender: ______________

Primary care provider name: ______________________________________ Phone: ________ Fax: ______________

☐ Patient does not have a primary care provider

	☐ Appointment #1 ☐ Appointment #2 ☐ Appointment #3 ☐ Appointment #4		
**Medical history**	Allergies, medical conditions, and medications are updated on patient record	☐ Yes	☐ No
	Age ≥ 18 years	☐ Yes	☐ No - STOP
	Documented allergy to tenofovir or emtricitabine	☐ Yes - STOP	☐ No
	Overlapping toxicities with other chronic medications*? *Recommend using **University of Liverpool HIV Drug Interaction Checker** (hyperlinked) to screen for interactions*	☐ Yes - STOP	☐ No
	Known eGFR < $60mL/min/1.73m^2$?	☐ Yes – STOP	☐ No
	Has a diagnosis of hepatitis B?	☐ Yes – STOP	☐ No
	Is pregnant or lactating?	☐ Yes - STOP	☐ No

**Laboratory results**	☐ Appointment #1 - laboratory results not applicable (*proceed to eligibility*)		
	**Appointment #2**		**Appointments #3 and #4**
	**HIV**: ☐ pos - STOP ☐ neg<br><br>**HIV viral load**:<br>☐ undetectable<br>☐ detectable – STOP<br><br>**HAV immunity**<br>☐ immune ☐ not immune<br><br>**HBV immunity (HBsAb)**<br>☐ immune ☐ not immune<br><br>**HBV infection (HBsAg):**<br>☐ pos - STOP ☐ neg	**Syphilis, chlamydia, gonorrhea:** ☐ pos – REFER for treatment, but continue ☐ neg<br><br>**eGFR**<br>☐ < $60 mL/min/1.73m^2$ - STOP<br>☐ > $60 mL/min/1.73m^2$<br><br>**Beta-HCG:** ☐ N/A<br>☐ pos – STOP ☐ neg<br><br>**ALT:** ☐ WNL	**HIV**: ☐ pos - STOP ☐ neg<br>**HIV viral load**:<br>☐ undetectable<br>☐ detectable – STOP<br><br>**eGFR**<br>☐ < $60 mL/min/1.73m^2$ - STOP<br>☐ > $60 mL/min/1.73m^2$<br><br>**Beta-HCG**: ☐ N/A<br>☐ pos - STOP ☐ neg<br><br>**ALT:** ☐ WNL<br>☐ > 54 U/L (male) or > 44 U/L (female) – STOP

	**HCV:**<br>☐ pos - STOP ☐ neg<br><br>**CBC**<br>☐ WNL<br>☐ abnormal - STOP	☐ > 54 U/L (male) or<br>> 44 U/L (female) – STOP<br><br>**Urinalysis**:<br>☐ WNL<br>☐ abnormal - STOP	**Syphilis, chlamydia, gonorrhea:**	☐ pos – REFER for treatment, but continue<br>☐ neg<br>☐ N/A (apt #3)

**Eligibility confirmation**<br><br>*as defined by Nova Scotia Pharmacare*	☐ Men who have sex with men (MSM) or transgender women (TGW)<br>• Reports condomless anal sex within the last 6 months **PLUS** any of the following:<br>○ Previous infectious syphilis or rectal bacterial STI<br>○ Recurrent use (>1) of nonoccupational postexposure prophylaxis (nPEP)<br>○ Ongoing sexual relationship with an HIV-positive partner not receiving stable ART and/or does not have an HIV viral load <200 copies/mL<br>○ High-incidence risk index (HIRI)-MSM risk score $\geq$ 11<br>Calculated HIRI-MSM risk score**:** ____
	☐ Heterosexual<br>• Reports condomless vaginal or anal sex **PLUS**<br>• Ongoing sexual relationship with an HIV-positive partner not receiving stable ART and/or does not have an HIV viral load <200 copies/mL
	☐ People who inject drugs (PWID)<br>• Reports sharing of injection equipment **PLUS**<br>• Have an HIV-positive injecting partner who is not receiving stable ART and/or does not have an HIV viral load <200 copies/mL
	Patient must meet one or more above criteria to be eligible.
**Assessment**	☐ The patient is eligible for PrEP (tenofovir/emtricitabine)<br>☐ The patient is NOT eligible for PrEP (tenofovir/emtricitabine) due to:<br>____<br>☐ The patient is referred to physician due to:<br>____
**Patient education**	☐ Patient education sheet reviewed with the following points discussed:<br>• Daily dosing of PrEP<br>• Importance of not missing doses<br>• Increase in HIV cases in Nova Scotia<br>• Potential adverse effects and management strategies<br>• N/V/D, flatulence<br>• Fatigue<br>• Headache<br>• ↓ in BMD<br>• ↓ in CrCl 1mL/min/year<br>• Laboratory monitoring protocols<br>• Patient self-monitoring protocols
**Follow-up**	☐ Initial appointment follow-up :<br>*Book next pharmacy appointment 3-7 days after laboratory appointment*<br>☐ *Halifax Sexual Health Centre patient information form completed and faxed*<br>☐ Confirmation and first prescribing appointment follow up date : ______________<br>*Next appointment at refill date (in 30 days)*

	☐ Routine prescribing appointment follow up date: ________________<br>*Next appointment at refill date*
**Laboratory requisitions**	• Initial appointment (appointment #1):<br>☐ Provide laboratory requisition 1 and assist patient with booking appointment online<br>• Confirmation and first prescribing appointment (appointment #2):<br>☐ Provide laboratory requisition 2 and assist patient with booking appointment online<br>☐ 30-day supply<br>• Routine prescribing appointment<br>☐ Provide laboratory requisition 3 and assist patient with booking appointment online, **AND**<br>☐ 60-day supply (appointment #3), *or*<br>☐ 90-day supply (appointment #4)<br>• Encourage anorectal and/or pharyngeal swab testing for chlamydia and gonorrhea every three months
**Immunization**	Encourage completion of applicable public health vaccines<br>• Individuals who use illicit drugs:<br>o Hepatitis A, hepatitis B, Pneu-P-23<br>• Men who have sex with men:<br>o Hepatitis A, hepatitis B, HPV<br>• All patients: routine COVID, TdaP, influenza
**Prescription**	Tenofovir disoproxil fumarate 300 mg and emtricitabine 200 mg po daily x<br>☐ 30 days (appointment #2)<br>☐ 60 days (appointment #3)<br>☐ 90 days (appointment #4)
**Pharmacist certification**	I have assessed the patient<br>• Patient consent was obtained<br>• The written prescription/assessment is within my scope of practice, skills, competencies, experience and is within the prescribing standards as outlined by the council.<br><br>Pharmacist Name: ____<br>Signature: ____<br>NSCP #: ______________<br>Date: ___________________________

HIV Incidence Risk Index for Men Who Have Sex With Men (HIRI-MSM) (Tan 2017)

1. How old are you today?
    a. If <18 years, 0 points STOP
    b. If 18-28 years, 8 points
    c. If 29-40 years, 5 points
    d. If 41-48 years, 2 points
    e. If ≥49 years, 0 points

2. In the last 6 months, how many men have you had sex with?
    a. If >10 male partners, 7 points
    b. If 6-10 male partners, 4 points
    c. If 0-5 male partners, 0 points

3. In the last 6 months, how many times did you have receptive anal sex (you were the bottom) with a man without a condom?
    a. If 1 or more times, 10 points
    b. If 0 times, 0 points

4. In the last 6 months, how many of your male sex partners were HIV-positive?
    a. If >1 positive partner, 8 points
    b. If 1 positive partner, 4 points
    c. If 0 positive partners, 0 points

5. In the last 6 months, how many times did you have insertive anal sex (you were the top) without a condom with a man who was HIV-positive?
    a. If 5 or more times, 6 points
    b. If <5 times, 0 points

6. In the last 6 months, have you used methamphetamines such as crystal or speed?
    a. If yes, 6 points
    b. If no, 0 points

Total Score____________

Prescription hard copy

Client information

Name: ____________________________ Preferred name/alias: ________________________
HCN: _____________________________
Street address: __________________________ City/town: _________________________
Province: ____________________________ Postal code: ___________
Phone number: _______________________
Date of birth: __________________________ Age: ___________
Gender: ____________________

Tenofovir disoproxil fumarate 300 mg and emtricitabine 200 mg po daily x

- ☐ 30 tabs (appointment #2)
- ☐ 60 tabs (appointment #3)
- ☐ 90 tabs (appointment #4)

No refills
Indication: HIV pre-exposure prophylaxis

☐ I, the assessing pharmacist, have seen and assessed the patient in person.

- The written prescription/assessment if any is within my scope of practice, skills, competencies, experience and is within the prescribing standards.

____ ________________________________

Pharmacist signature/license # Date

Fax cover letter

To:	Fax:
From:	Pages:
Phone:	Date
Re:	cc:

Comments

Physician/NP prescribing notification

☐ Response required ☐ For your records

**Client information**
Name: ____________________________ Preferred name/alias: ________________________<br>HCN: _____________________________<br>Street address: __________________________ City/town: _________________________<br>Province: ____________________________ Postal code: ___________<br>Phone number: _______________________<br>Date of birth: __________________________ Age: ___________<br>Gender: ____________________________<br>Primary care provider name: ________________________________PCP phone #:______________________<br>Primary care provider fax number: _______________________________<br>Primary care provider address:<br>____

Your patient has been assessed and prescribed for HIV pre-exposure prophylaxis as part of the PrEP-Rx study being conducted by Dr. Kyle Wilby at Dalhousie University. The patient was prescribed the following therapy:

Tenofovir disoproxil fumarate 300 mg and emtricitabine 200 mg po daily<br>☐ 30 tabs (appointment #2)<br>☐ 60 tabs (appointment #3)<br>☐ 90 tabs (appointment #4)<br><br>No refills
The following Lab Test Requisitions were provided as per an approved protocol through a collaborative practice framework with the Halifax Sexual Health Centre under the authority of Dr. Matthew Lee.<br><br>Results from the last requisition were ________
Follow-up plan:

I, the assessing pharmacist, have seen and assessed the patient in-person.

- The written prescription/assessment if any is within my scope of practice, skills, competencies, experience and is within the prescribing standards.

____ ____

Pharmacist signature/license # Date

d'Entremont-Harris M, Ramsey TD, MacNabb K, et al. Implementation and acceptance of pharmacists' prescribing of Human Immunodeficiency Virus (HIV) Pre-Exposure Prophylaxis (PrEP). Can Pharm J (Ott) 2025;158. DOI 10.1177/17151635231355277.
